# Longitudinal trajectories of the triglyceride-glucose index and risk of incident type 2 diabetes in adults undergoing repeated health examinations

**DOI:** 10.3389/fendo.2026.1895734

**Published:** 2026-08-03

**Authors:** Nanping Mao, Ruoli Wang, Yi Wang, Lingna Jin, Yanyun He, Wenbin Wu

**Affiliations:** 1 Hospital of Chengdu University of Traditional Chinese Medicine, Chengdu, Sichuan, China; 2 Health Management Center, Hospital of Chengdu University of Traditional Chinese Medicine, Chengdu, Sichuan, China; 3 Department of Geriatrics, Hospital of Chengdu University of Traditional Chinese Medicine, Chengdu, Sichuan, China

**Keywords:** insulin resistance, landmark analysis, longitudinal cohort, risk stratification, triglyceride-glucose index, TyG trajectory, type 2 diabetes mellitus

## Abstract

**Background:**

The triglyceride-glucose (TyG) index is an established surrogate marker of insulin resistance. However, most evidence has relied on a single baseline measurement and may not fully capture long-term metabolic exposure. This study examined whether longitudinal TyG trajectories are independently associated with subsequent incident type 2 diabetes mellitus (T2DM).

**Methods:**

This retrospective longitudinal cohort study used repeated health examination data from adults without diabetes at baseline between 2011 and 2025. TyG trajectories were identified using latent class mixed models based on repeated TyG measurements. Follow-up for incident T2DM began after the third eligible TyG measurement to preserve temporal ordering between exposure assessment and outcome occurrence. Multivariable Cox proportional hazards models with multiple imputation were used to estimate hazard ratios (HRs) and 95% confidence intervals (CIs). Restricted cubic splines and time-dependent area under the curve (AUC) analyses were used for dose-response and predictive performance assessments.

**Results:**

Among 16, 751 participants included in trajectory modeling, three TyG trajectory groups were identified and ordered by mean TyG level: low-ascending (G1), moderate-ascending (G2), and high-persistent (G3). A total of 10, 163 participants contributed follow-up after the third-visit landmark, among whom 430 incident T2DM events were recorded. Incidence rates were 2.90, 13.47, and 37.25 per 1, 000 person-years in G1, G2, and G3, respectively. In the main adjusted model, compared with G1, both G2 (HR = 2.27, 95% CI: 1.64-3.14) and G3 (HR = 5.40, 95% CI: 3.87-7.53) were associated with higher risks of incident T2DM (both P < 0.001). A graded association was observed across trajectory levels (HR per level increase = 2.34, 95% CI: 2.01-2.72; P < 0.001). Adding TyG information provided modest incremental discrimination beyond the base clinical model, with limited additional improvement over baseline TyG alone.

**Conclusions:**

Longitudinal TyG trajectories identified distinct diabetes risk strata among adults undergoing repeated health examinations. A high-persistent TyG trajectory was associated with increased risk of incident T2DM after the landmark time point, supporting the potential value of monitoring TyG dynamics for early risk stratification in routine care.

## 1 Introduction

Type 2 diabetes mellitus (T2DM) remains a major global health burden (1, 2). Insulin resistance (IR) is central to T2DM development, making early identification of metabolic risk important for prevention (3, 4). The hyperinsulinemic-euglycemic clamp is the reference method for assessing IR but is impractical for routine or large-scale use (5). The triglyceride-glucose (TyG) index, calculated from fasting triglyceride and glucose concentrations, is a simple surrogate marker of IR (6, 7) and has been associated with incident T2DM (8) and other cardiometabolic outcomes (9–11).

Insulin resistance is also linked to broader cardiometabolic disorders, including obesity, vascular dysfunction, cardiovascular disease, and kidney disease (12). Most evidence on the prognostic value of the TyG index has relied on a single baseline measurement (13). A baseline TyG value is useful for initial risk assessment, but it may be affected by short-term diet, physical activity, medication use, or acute physiological stress and may not adequately represent cumulative metabolic exposure during the preclinical phase of T2DM.

Longitudinal trajectory analysis can characterize repeated exposure patterns over time and may provide information beyond a single measurement (14). Recent studies have examined TyG trajectories in relation to frailty, cardio-renal-metabolic multimorbidity, and stroke (15–17), but evidence linking TyG trajectory patterns to incident T2DM remains limited.

To address this gap, we used repeated health examination data from 2011 to 2025 to identify TyG trajectory patterns before a prespecified landmark time and to evaluate their associations with subsequent incident T2DM.

## 2 Methods

### 2.1 Study design and data source

This retrospective longitudinal cohort study used electronic medical records from a comprehensive health examination center between 2011 and 2025. The database included repeated health examination records, questionnaire data, anthropometric measurements, blood pressure, and routinely measured biochemical indices. The analysis included adults with repeated examinations and available triglyceride-glucose (TyG)-related laboratory data.

### 2.2 Study population and landmark cohort

**Figure 1:**
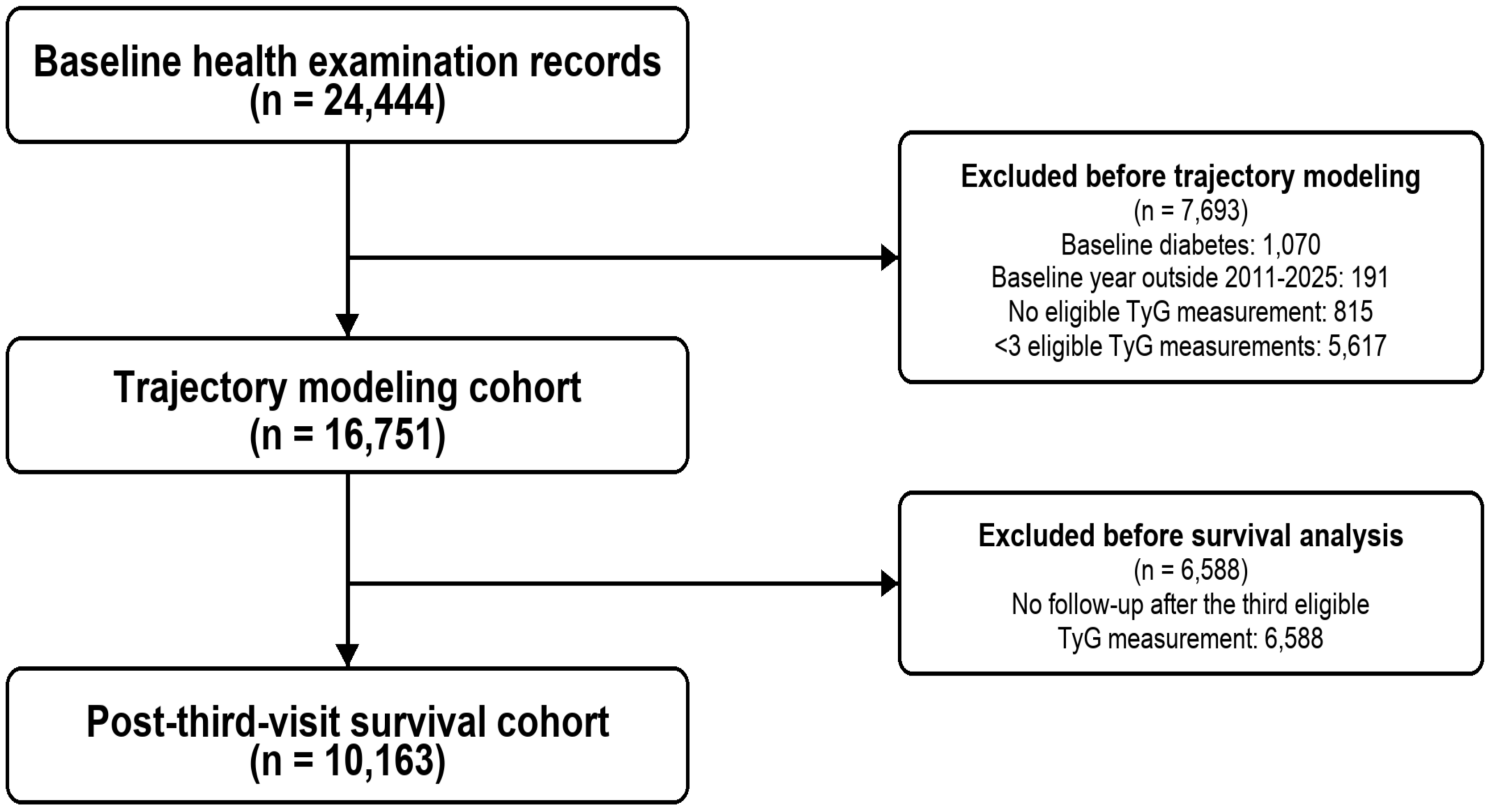
Flowchart of the study population selection. The flowchart shows the inclusion and exclusion process from baseline records to the trajectory modeling cohort and the post-third-visit survival cohort.

Among 24, 444 baseline health examination records, participants were eligible if they were aged 18 years or older, had no diabetes at baseline, had a baseline examination between 2011 and 2025, had valid identification and date information, and had at least three eligible TyG measurements before diabetes onset or censoring. A total of 16, 751 participants were included in trajectory modeling (**Supplementary Table 1**). For each participant, the first three eligible TyG measurements were used to define the early TyG trajectory. Follow-up for incident T2DM started after the third eligible TyG measurement. Participants without any follow-up after this point were excluded from survival analyses, leaving 10, 163 participants in the post-landmark cohort (**Figure 1**; **Supplementary Table 2**).

### 2.3 Construction of the analytic dataset

Repeated TyG measurements were arranged in long format using years since the first eligible examination as the time scale. Only TyG measurements obtained before diabetes onset or censoring were eligible. In the primary analysis, trajectory groups were assigned using TyG measurements up to and including the third eligible measurement, which served as the landmark time point, and diabetes events were evaluated only after that time point. Participants who developed T2DM before or at the third eligible TyG measurement, or who had no eligible follow-up after that visit, were not included in the post-landmark survival analysis. This landmark design reduced reverse causation and prevented TyG values measured during outcome follow-up from being used to define trajectory groups.

### 2.4 TyG calculation

The TyG index was calculated from fasting triglycerides and fasting glucose as ln [triglycerides (mg/dL) × glucose (mg/dL) / 2]. Values measured in mmol/L were converted to mg/dL before calculation. To reduce the influence of extreme values while retaining all participants, TyG values below the 0.5th percentile were set to the 0.5th percentile and values above the 99.5th percentile were set to the 99.5th percentile.

### 2.5 TyG trajectory modeling

TyG trajectories were modeled using latent class mixed models implemented with the lcmm package. Candidate models with one to four latent classes were compared using AIC, BIC, log likelihood, minimum class proportion, average posterior probability, entropy, convergence, and clinical interpretability. Although the four-class model had a lower BIC, it generated a very small class (0.73% of the trajectory cohort) and was less clinically distinguishable. Therefore, the three-class solution was selected to balance model fit, classification stability, and interpretability (**Supplementary Table 4**).

### 2.6 Outcome ascertainment

Baseline diabetes was identified from the diabetes history recorded at the baseline health examination or a baseline fasting plasma glucose level ≥7.0 mmol/L. Incident T2DM was identified when diabetes was first recorded during follow-up or when fasting plasma glucose first reached ≥7.0 mmol/L. The event date was defined as the date of the first health examination meeting either criterion. Participants who remained free of T2DM were censored at their last available health examination, with follow-up extending through 2025.

### 2.7 Covariates

Covariates were taken from each participant’s first eligible health examination. These included age, sex, body mass index (BMI), systolic blood pressure (SBP), diastolic blood pressure (DBP), uric acid, and lipid indices. Although diabetes follow-up began after the third eligible TyG measurement, baseline covariates were used in **Table 1** and in the multivariable Cox models. This avoided adjusting for measurements that may have changed during the period used to define TyG trajectories.

**Table 1:** Baseline characteristics at the first eligible examination among participants in the post-landmark survival cohort, according to TyG trajectory group.

Variable	Overall	G1	G2	G3	P value
n	10163	4272	4159	1732	
Age, years	43.73 (16.29)	37.84 (14.75)	47.97 (16.45)	48.10 (14.93)	<0.001
Sex, Male n (%)	4202 (41.3)	1042 (24.4)	2022 (48.6)	1138 (65.7)	<0.001
BMI, kg/m2	22.89 (3.22)	21.26 (2.68)	23.63 (2.99)	25.04 (3.02)	<0.001
SBP, mmHg	119.10 (17.31)	113.06 (14.92)	122.46 (17.65)	125.96 (17.25)	<0.001
DBP, mmHg	73.27 (10.56)	70.40 (9.40)	74.38 (10.62)	77.65 (11.10)	<0.001
Total cholesterol, mmol/L	4.57 (0.90)	4.22 (0.79)	4.74 (0.87)	5.01 (0.93)	<0.001
Triglycerides, mmol/L	1.42 (1.00)	0.81 (0.25)	1.42 (0.43)	2.89 (1.48)	<0.001
LDL-C, mmol/L	2.79 (0.84)	2.40 (0.68)	3.04 (0.81)	3.12 (0.88)	<0.001
HDL-C, mmol/L	1.44 (0.37)	1.62 (0.35)	1.37 (0.33)	1.14 (0.26)	<0.001
Uric acid, umol/L	331.10 (90.11)	293.26 (72.04)	343.99 (86.78)	393.62 (94.51)	<0.001
Baseline TyG	8.40 (0.57)	7.92 (0.31)	8.54 (0.30)	9.23 (0.43)	<0.001

All characteristics were measured at the first eligible examination, whereas follow-up for survival analyses began after the third eligible TyG measurement. Continuous variables are presented as mean (SD), and categorical variables are presented as n (%). P values compare baseline characteristics across TyG trajectory groups.

TyG, triglyceride-glucose; G1, low-ascending trajectory; G2, moderate-ascending trajectory; G3, high-persistent trajectory; SD, standard deviation; BMI, body mass index; SBP, systolic blood pressure; DBP, diastolic blood pressure; TC, total cholesterol; TG, triglycerides; LDL-C, low-density lipoprotein cholesterol; HDL-C, high-density lipoprotein cholesterol.

### 2.8 Statistical analysis

Continuous variables are presented as mean (standard deviation, SD) or median (interquartile range, IQR), and categorical variables are presented as counts and percentages. Baseline characteristics were compared across trajectory groups using appropriate parametric or non-parametric tests for continuous variables and chi-square tests for categorical variables. Missing covariate values were imputed using multiple imputation by chained equations with five imputed datasets; missingness and imputation methods are summarized in **Supplementary Table 3**.

Cox proportional hazards models were used to estimate hazard ratios (HRs) and 95% confidence intervals (CIs) for incident T2DM after the landmark time. Model 1 was unadjusted. Model 2 adjusted for baseline age and sex. Model 3 further adjusted for baseline BMI, SBP, DBP, and uric acid and was considered the main model. Because lipid variables, especially triglycerides, are biologically related to the TyG index, additional adjustment for cholesterol (TC), triglycerides (TG), low-density lipoprotein cholesterol (LDL-C), and high-density lipoprotein cholesterol (HDL-C) was treated as a metabolic sensitivity analysis rather than as the primary model (**Supplementary Table 5**).

Sensitivity analyses included complete-case analysis, baseline TyG quartiles, exclusion of events within 1 year after the landmark, and exclusion of participants with fasting plasma glucose ≥6.1 mmol/L at the landmark visit (**Supplementary Table 5**). The proportional hazards assumption was assessed using Schoenfeld residuals, and 5-year restricted mean survival time(RMST) was calculated as a complementary analysis. Restricted cubic splines and time-dependent AUCs were used for dose-response and predictive performance analyses. Prespecified subgroup analyses were exploratory and are summarized in **Supplementary Table 6**. A two-sided P value < 0.05 was considered statistically significant.

## 3 Results

### 3.1 Baseline characteristics, cohort flow, and TyG trajectories

**Figure 2:**
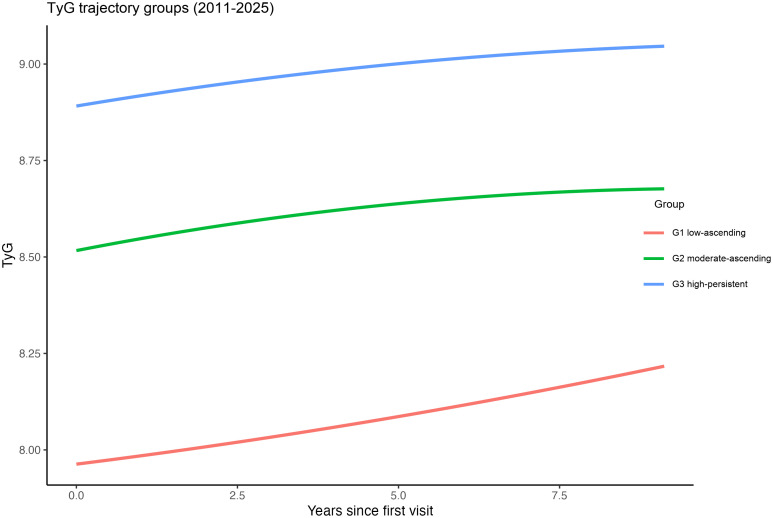
Identification of triglyceride-glucose (TyG) index trajectory groups.

A total of 16, 751 participants were included in trajectory modeling, of whom 10, 163 had eligible follow-up after the third TyG measurement and were included in the primary survival analysis (**Figure 1**). Based on LCMM analysis of repeated TyG measurements before the landmark time point, three trajectory groups were identified and ordered according to mean TyG level: low-ascending (G1), moderate-ascending (G2), and high-persistent (G3) (**Figure 2**). In the trajectory modeling cohort, the group sizes were 6, 763, 6, 976, and 3, 012 for G1, G2, and G3, respectively. In the post-landmark cohort, the corresponding group sizes were 4, 272, 4, 159, and 1, 732.

Baseline characteristics measured at the first eligible examination among participants in the post-landmark cohort are shown in **Table 1**. Participants in higher TyG trajectory groups were more likely to be male and had less favorable baseline cardiometabolic profiles, including higher BMI, SBP, DBP, TC, TG, uric acid, and baseline TyG, as well as lower HDL-C levels (all P < 0.001).

### 3.2 Longitudinal association between TyG trajectories and incident T2DM

**Figure 3:**
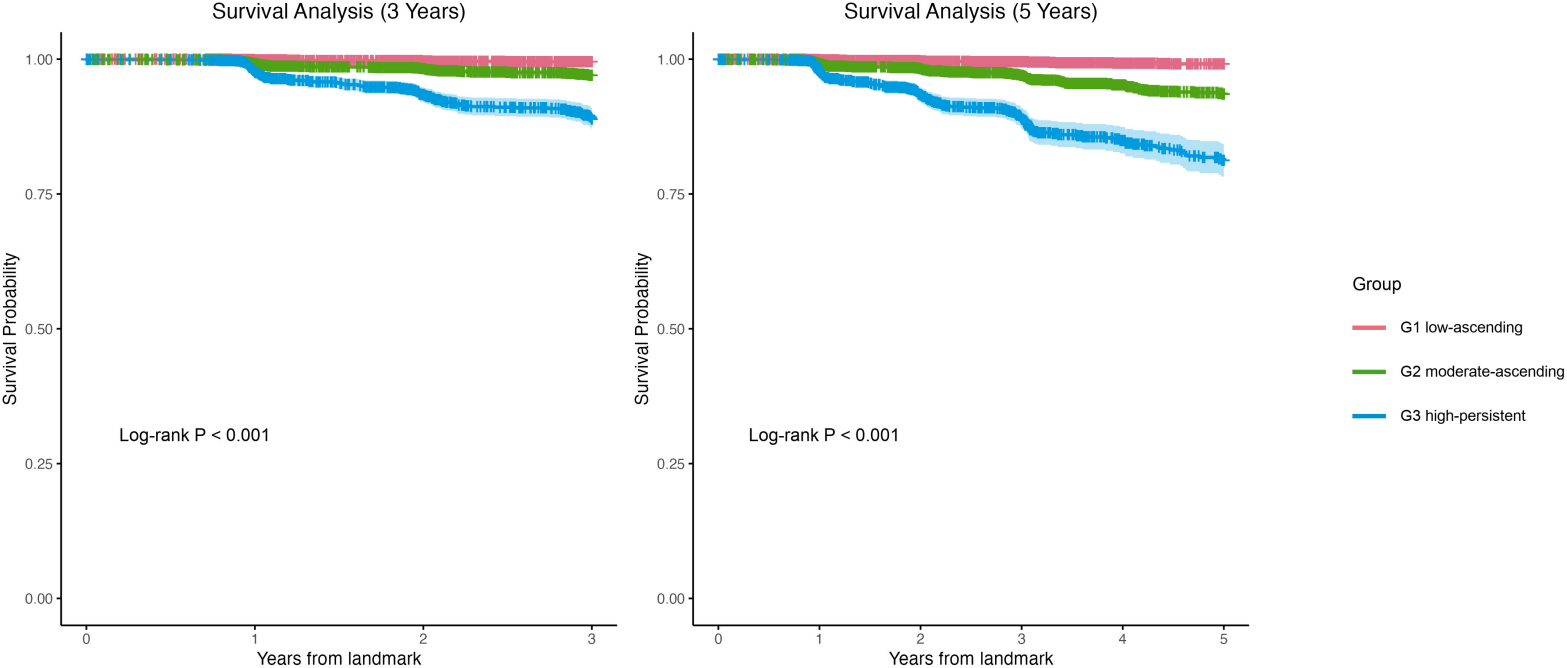
Kaplan-Meier survival curves for incident T2DM by TyG trajectory group. Kaplan-Meier curves show diabetes-free survival after the third-visit landmark, stratified by TyG trajectory group. Differences between groups were assessed using the log-rank test.

After the third eligible TyG measurement, 430 participants developed incident T2DM during subsequent follow-up. The incidence rate increased stepwise across TyG trajectory groups, from 2.90 per 1, 000 person-years in G1 to 13.47 per 1, 000 person-years in G2 and 37.25 per 1, 000 person-years in G3 (**Table 2**). Kaplan-Meier curves showed lower diabetes-free survival in the higher TyG trajectory groups (**Figure 3**).

**Table 2:** Event summary by TyG trajectory group.

Trajectory group	N	Events	Event rate, %	Person-years	Incidence per 1, 000 PY	Follow-up, median (IQR), years
G1	4272	50	1.17	17218.5	2.9	2.90 (1.24, 5.91)
G2	4159	183	4.4	13585.4	13.47	2.11 (1.02, 3.96)
G3	1732	197	11.37	5288.5	37.25	2.05 (1.04, 3.54)

Events, person-years, crude event rates, incidence rates, and follow-up distributions are summarized from the third-visit landmark. Incidence rates are expressed per 1, 000 person-years.

TyG, triglyceride-glucose; PY, person-years; IQR, interquartile range; G1, low-ascending trajectory; G2, moderate-ascending trajectory; G3, high-persistent trajectory.

These findings were confirmed in Cox regression analyses (**Table 3**). In the fully adjusted Model, compared with G1, the HRs for incident T2DM were 2.27 (95% CI: 1.64-3.14; P < 0.001) for G2 and 5.40 (95% CI: 3.87-7.53; P < 0.001) for G3, with a significant graded association across trajectory levels (HR per trajectory-level increase = 2.34, 95% CI: 2.01-2.72; P < 0.001). The associations were materially unchanged after additional lipid adjustment and remained significant after excluding participants with fasting plasma glucose ≥6.1 mmol/L at the landmark visit, although the effect estimates were attenuated (**Supplementary Table 5**). Because Schoenfeld residual tests indicated nonproportional hazards for trajectory group (**Supplementary Table 7**), we performed complementary 5-year RMST analyses, which showed progressively shorter diabetes-free survival across G1, G2, and G3 (4.98, 4.86, and 4.56 years, respectively); compared with G1, the RMST differences were -0.12 years for G2 and -0.42 years for G3 (**Supplementary Table 8**). Restricted cubic spline analyses did not show clear evidence of nonlinear associations within trajectory groups (all P for nonlinearity >0.30; **Supplementary Table 10**).

**Table 3:** Association between TyG trajectory group and incident T2DM from pooled Cox proportional hazards models.

TyG trajectory group	Model 1 HR (95% CI)	Model 2 HR (95% CI)	Model 3 HR (95% CI)
G1 low-ascending	Reference	Reference	Reference
G2 moderate-ascending	4.91 (3.59-6.72)	2.85 (2.07-3.92)	2.27 (1.64-3.14)
G3 high-persistent	13.99 (10.24-19.11)	8.02 (5.84-11.01)	5.40 (3.87-7.53)
Per trajectory-level increase	3.46 (3.03-3.96)	2.82 (2.45-3.26)	2.34 (2.01-2.72)

Model 1 was unadjusted. Model 2 adjusted for baseline age and sex. Model 3 adjusted for baseline age, sex, BMI, SBP, DBP, and uric acid. TyG, triglyceride-glucose; HR, hazard ratio; CI, confidence interval; BMI, body mass index; SBP, systolic blood pressure; DBP, diastolic blood pressure. P for trend < 0.001 for Models 1-3.

### 3.3 Subgroup analysis and time-dependent predictive performance

**Figure 4:**
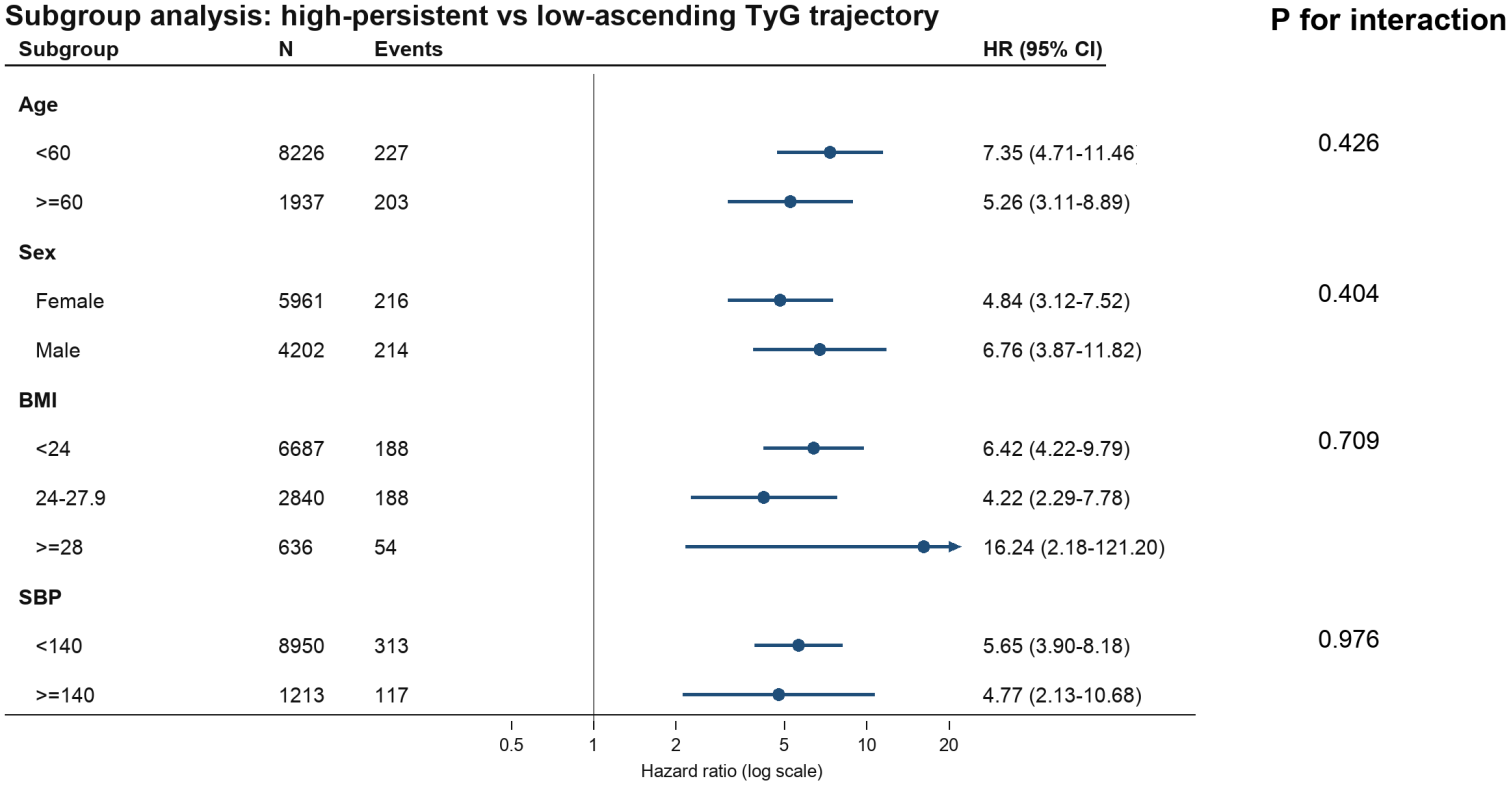
Subgroup analysis for the association between TyG trajectories and incident T2DM. The forest plot shows subgroup-specific HRs and 95% CIs for incident T2DM comparing the high-persistent trajectory group with the low-ascending trajectory group across prespecified strata.

Subgroup analyses showed generally consistent associations across strata of sex, age, BMI, and blood pressure, with no clear evidence of interaction (**Figure 4**; **Supplementary Table 6**).

**Figure 5:**
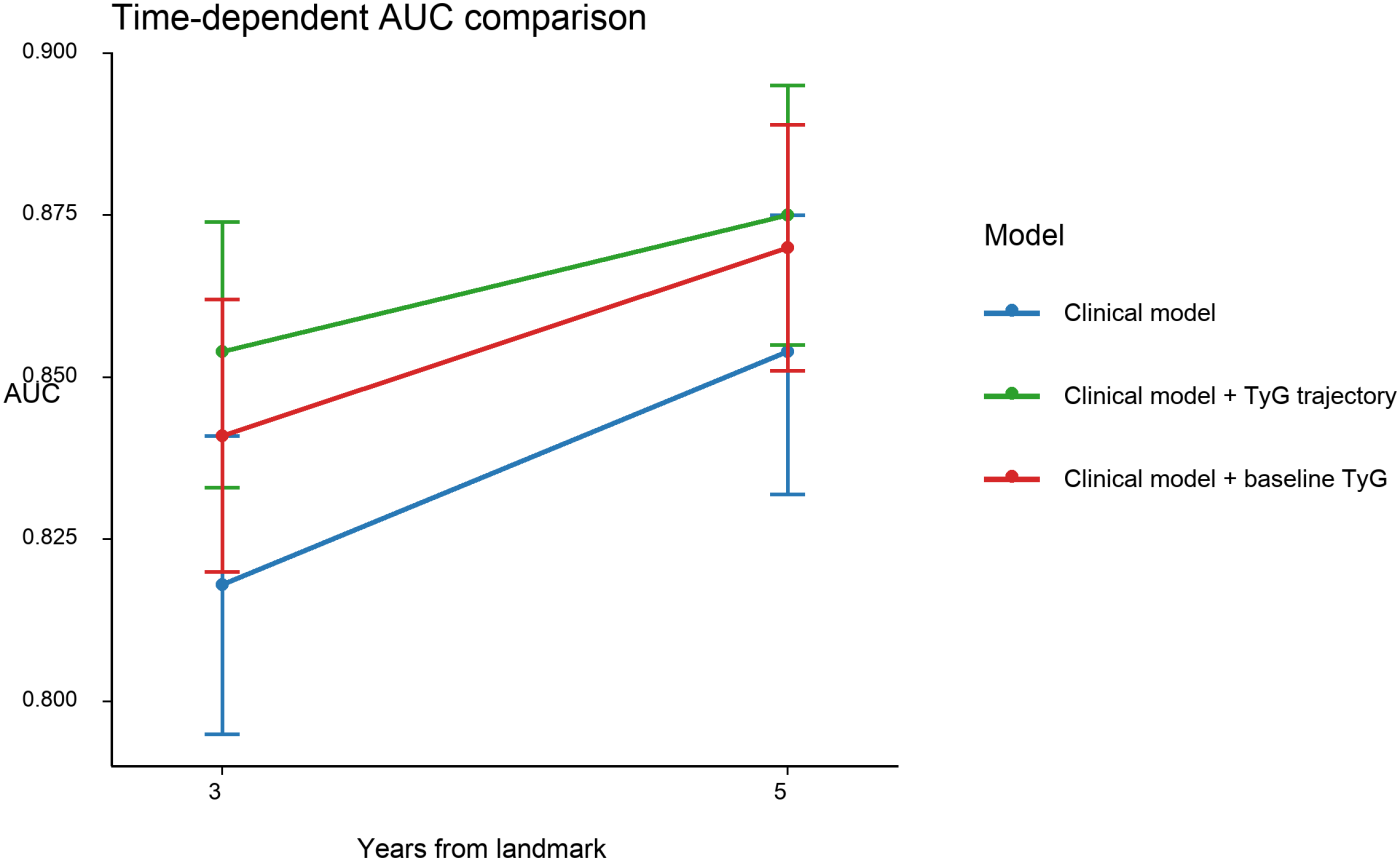
Time-dependent AUC comparison across prediction models. Time-dependent AUCs are shown for the base clinical model, the base model plus baseline TyG, and the base model plus TyG trajectory group at 3 and 5 years after the landmark. AUC, area under the curve; TyG, triglyceride-glucose.

We further evaluated time-dependent predictive performance using model-based AUCs (**Table 4**; **Figure 5**). At 3 years, the AUC was 0.818 (95% CI: 0.795-0.841) for the base clinical model, 0.841 (95% CI: 0.820-0.862) for the base model plus baseline TyG, and 0.854 (95% CI: 0.833-0.874) for the base model plus TyG trajectory group. At 5 years, the corresponding AUCs were 0.854 (95% CI: 0.832-0.875), 0.870 (95% CI: 0.851-0.889), and 0.875 (95% CI: 0.855-0.895). Thus, TyG information provided modest incremental discrimination beyond the base clinical model; the trajectory-based model showed limited additional gain over baseline TyG. Formal AUC comparisons showed that the trajectory model improved discrimination compared with the base clinical model at 3 and 5 years, whereas the additional improvement over baseline TyG alone was small and was not statistically significant at 5 years (**Supplementary Table 9**). Ten-year prediction was not estimated because follow-up after the landmark time point was insufficient.

**Table 4:** Time-dependent AUC comparison across prediction models.

Model	3-year AUC (95% CI)	5-year AUC (95% CI)
Clinical model	0.818 (0.795-0.841)	0.854 (0.832-0.875)
Clinical model + baseline TyG	0.841 (0.820-0.862)	0.870 (0.851-0.889)
Clinical model + TyG trajectory	0.854 (0.833-0.874)	0.875 (0.855-0.895)

The clinical model included baseline age, sex, BMI, SBP, DBP, and uric acid. Baseline TyG and TyG trajectory group were entered separately into the clinical model. Time-dependent AUCs were estimated at 3 and 5 years from the landmark time defined at the third eligible visit.

AUC, area under the curve; CI, confidence interval; TyG, triglyceride-glucose.

## 4 Discussion

In this large-scale longitudinal cohort study, we identified three distinct TyG trajectory groups and evaluated their associations with incident T2DM. Participants with moderate-ascending and high-persistent TyG trajectories had higher risks of incident T2DM than those with a low-ascending trajectory. These associations persisted after adjustment for demographic, anthropometric, blood pressure, and biochemical covariates, with a clear graded trend across trajectory groups. By defining trajectories before the start of outcome follow-up, the analysis reduced the risk that later measurements influenced exposure classification.

Previous studies have shown that a higher baseline TyG index is associated with increased risks of T2DM and adverse cardiometabolic outcomes (18–21). Large cohort studies in Chinese adults have reported substantially higher diabetes risk among individuals with elevated TyG levels (22, 23), and other studies have suggested nonlinear or threshold associations between TyG and diabetes susceptibility (24). However, a single TyG measurement may be influenced by short-term factors and may not capture cumulative metabolic exposure (17, 25). Trajectory-based approaches can summarize repeated exposure patterns and may better reflect persistent metabolic stress over time. Nevertheless, trajectory classification should be interpreted with caution and supported by transparent model selection and clinical interpretability (21). In the present study, three trajectory groups provided a clinically meaningful low-to-high metabolic gradient with acceptable classification quality.

The observed association is biologically plausible. The TyG index reflects the joint burden of fasting glucose and triglycerides and is commonly used as a surrogate marker of IR. Sustained elevations in glucose and triglycerides may indicate chronic hepatic and skeletal muscle insulin resistance, ectopic lipid accumulation, and glucolipotoxic stress (26–28). Increased free fatty acid flux can impair insulin signaling and worsen systemic insulin resistance (29, 30). Over time, compensatory insulin hypersecretion may increase beta-cell workload, whereas glucolipotoxicity, oxidative stress, endoplasmic reticulum stress, and low-grade inflammation may contribute to beta-cell dysfunction and progression to overt diabetes (31–34). Because these mechanisms were not directly measured in this study, they should be viewed as biologically plausible explanations for the observed epidemiological association rather than mechanistic evidence.

From a clinical perspective, TyG trajectory monitoring is practical because fasting glucose and triglycerides are routinely measured in health examination and primary care settings. The incidence rates in this study showed a clear gradient across trajectory groups, suggesting that repeated TyG assessment may help identify individuals at higher metabolic risk. Sensitivity analyses, including complete-case results, baseline TyG quartiles, exclusion of events within 1 year and exclusion of participants with fasting plasma glucose ≥6.1 mmol/L at the landmark visit, generally supported the main findings. Nevertheless, the incremental predictive value of trajectory monitoring was modest and should be viewed as clinically complementary rather than as a replacement for baseline assessment.

Several limitations should be noted. First, this was an observational study, so causal inference is limited despite the landmark design. Second, requiring at least three eligible TyG measurements may have selected participants who attended health examinations more regularly. In addition, 6, 588 participants included in trajectory modeling were not retained in the post-landmark survival cohort because they had no eligible follow-up after the third TyG measurement; these participants differed from retained participants in several baseline characteristics, suggesting potential attrition bias. Third, because TyG includes fasting glucose and incident T2DM was mainly defined using fasting glucose, part of the observed association may reflect shared measurement components. Although the association persisted after excluding participants with fasting plasma glucose ≥6.1 mmol/L at the landmark visit, definitional overlap cannot be fully excluded. Fourth, diabetes ascertainment relied on available examination and medical record data; HbA1c, oral glucose tolerance testing, ICD codes, medication details, diabetes subtype, and repeated confirmatory testing were not uniformly available. Fifth, residual confounding from lifestyle factors, family history, medication changes, and detailed adiposity measures could not be excluded. Finally, subgroup analyses were exploratory because some strata contained few events, and the proportional hazards assumption was not fully met for trajectory group; therefore, Cox estimates were interpreted as average associations across follow-up. Complementary RMST analyses supported a graded reduction in diabetes-free survival time across trajectory groups.

## 5 Conclusion

Longitudinal TyG trajectories were associated with subsequent T2DM risk among adults undergoing repeated health examinations. These findings suggest that TyG trajectory monitoring may provide complementary risk-stratification information, although its incremental predictive value beyond baseline TyG appears limited.

## Data Availability

Further inquiries can be directed to the corresponding authors. Requests to access these datasets should be directed to wwb1201@vip.sina.com.
