# Supplementary material for: Longitudinal trajectories of the triglyceride-glucose index and risk of incident type 2 diabetes in adults undergoing repeated health examinations
Source: Front Endocrinol (Lausanne). 2026 Aug 3;17:1895734. doi: 10.3389/fendo.2026.1895734 (PMC13477973; doi:10.3389/fendo.2026.1895734)
Supplement: Supplementary file 1 [file DataSheet1.pdf]

Table S1. Baseline characteristics of participants included in and excluded from trajectory modeling.

Variable	Included	Excluded	P value
n	16751	5617	
Age, years	43.11 (15.77)	39.89 (14.52)	<0.001
Sex, Male n (%)	7204 (43.0)	2615 (46.6)	<0.001
BMI, kg/m2	23.06 (3.26)	23.30 (3.52)	<0.001
SBP, mmHg	119.04 (17.12)	118.85 (16.33)	0.484
DBP, mmHg	72.92 (10.66)	73.39 (10.68)	0.006
Total cholesterol, mmol/L	4.57 (0.89)	4.55 (0.87)	0.236
Triglycerides, mmol/L	1.45 (1.06)	1.56 (1.33)	<0.001
LDL-C, mmol/L	2.84 (0.84)	2.72 (0.77)	<0.001
HDL-C, mmol/L	1.38 (0.36)	1.37 (0.35)	0.236
Uric acid, umol/L	336.53 (91.29)	345.08 (93.34)	<0.001
Baseline TyG	8.42 (0.58)	8.47 (0.64)	<0.001
Creatinine, umol/L	66.87 (17.77)	66.13 (18.85)	0.008

Note: Included participants had at least three eligible TyG measurements for trajectory modeling; excluded participants had fewer than three eligible TyG measurements. Continuous variables are presented as mean (SD), and categorical variables are presented as n (%).

Table S2. Baseline characteristics of participants retained in and excluded from the post-landmark survival cohort.

Variable	Excluded	Retained	P value
n	6,588	10,163	
Age, years	42.15 (14.90)	43.73 (16.29)	<0.001
Sex, Male n (%)	3,002 (45.6)	4,202 (41.3)	<0.001
BMI, kg/m2	23.31 (3.31)	22.89 (3.22)	<0.001
SBP, mmHg	118.93 (16.83)	119.10 (17.31)	0.544

Variable	Excluded	Retained	P value
DBP, mmHg	72.40 (10.78)	73.27 (10.56)	<0.001
Total cholesterol, mmol/L	4.57 (0.86)	4.57 (0.90)	0.779
Triglycerides, mmol/L	1.49 (1.14)	1.42 (1.00)	<0.001
LDL-C, mmol/L	2.92 (0.85)	2.79 (0.84)	<0.001
HDL-C, mmol/L	1.30 (0.33)	1.44 (0.37)	<0.001
Uric acid, umol/L	344.89 (92.48)	331.10 (90.11)	<0.001
Baseline TyG	8.46 (0.58)	8.40 (0.57)	<0.001
Creatinine, umol/L	66.66 (15.65)	67.00 (19.02)	0.219

Note: Excluded participants were those included in trajectory modeling but not retained in the post-landmark survival cohort because they had no eligible follow-up after the third eligible TyG measurement. Continuous variables are presented as mean (SD), and categorical variables are presented as n (%).

Table S3. Missingness and multiple-imputation methods.

Variable	Missing, %	Imputation method
Baseline TyG	11.8	pmm
Total cholesterol	11.6	pmm
HDL-C	11.6	pmm
Triglycerides	11.5	pmm
LDL-C	11.5	pmm
BMI	7.1	pmm
DBP	4.8	pmm
SBP	4.8	pmm
Uric acid	0.3	pmm
Age	0.0	Not imputed
Sex	0.0	Not imputed

Note: pmm indicates predictive mean matching. Variables with no missing data were not imputed. Event and follow-up variables were not imputed.

Table S4. Latent trajectory model comparison.

Classes	AIC	BIC	Log likelihood	Minimum class, %	Average posterior probability	Entropy
1	54598.1	54652.2	-27292.0	100.00	1.000	1.000
2	53643.3	53736.0	-26809.6	47.26	0.775	0.329
3	53401.4	53532.7	-26683.7	17.98	0.707	0.392
4	52911.4	53081.4	-26433.7	0.73	0.761	0.629

Note: The three-class solution was selected to balance fit, classification stability, and clinical interpretability.

Table S5. Sensitivity analyses for TyG-related exposures and incident T2DM.

Analysis	Exposure category	HR (95% CI)	P value
Complete-case analysis	G1 low-ascending	Reference	-
Complete-case analysis	G2 moderate-ascending	2.19 (1.56-3.08)	<0.001
Complete-case analysis	G3 high-persistent	5.13 (3.62-7.27)	<0.001
Baseline TyG quartiles	Quartile 1	Reference	-
Baseline TyG quartiles	Quartile 2	2.16 (1.25-3.74)	0.006
Baseline TyG quartiles	Quartile 3	3.35 (2.00-5.60)	<0.001
Baseline TyG quartiles	Quartile 4	5.61 (3.38-9.31)	<0.001
Excluding events within 1 year	G1 low-ascending	Reference	-
Excluding events within 1 year	G2 moderate-ascending	2.16 (1.53-3.05)	<0.001
Excluding events within 1 year	G3 high-persistent	5.13 (3.60-7.30)	<0.001
Excluding landmark FPG ≥6.1 mmol/L	G1 low-ascending	Reference	-
Excluding landmark FPG ≥6.1 mmol/L	G2 moderate-ascending	2.14 (1.53-3.00)	<0.001
Excluding landmark FPG ≥6.1 mmol/L	G3 high-persistent	4.26 (2.99-6.08)	<0.001
Metabolic sensitivity model	G1 low-ascending	Reference	-
Metabolic sensitivity model	G2 moderate-ascending	2.23 (1.59-3.13)	<0.001
Metabolic sensitivity model	G3 high-persistent	5.85 (3.89-8.78)	<0.001

Note: G1 low-ascending and Quartile 1 were used as reference categories. The complete-case analysis included 9,400 participants and 389 events; the analysis excluding events within 1 year included 10,091 participants and 358 events; and the analysis excluding participants with fasting plasma glucose ≥6.1 mmol/L at the landmark visit included 9,954 participants and 345 events. The metabolic sensitivity model additionally adjusted for TC, TG, LDL-C, and HDL-C and was interpreted cautiously because these lipid variables are biologically related to TyG. Abbreviations: CI, confidence interval; FPG, fasting plasma glucose; HDL-C, high-density lipoprotein cholesterol; HR, hazard ratio; LDL-C, low-density lipoprotein cholesterol; TC, total cholesterol; TG, triglycerides; TyG, triglyceride-glucose.

Table S6. Subgroup analyses and interaction tests.

Subgroup	Level	N	Events	HR (95% CI)	P value	P interaction
Age	<60	8226	227	7.35 (4.71-11.46)	<0.001	0.426
Age	>=60	1937	203	5.26 (3.11-8.89)	<0.001	
Sex	Female	5961	216	4.84 (3.12-7.52)	<0.001	0.404
Sex	Male	4202	214	6.76 (3.87-11.82)	<0.001	
BMI	<24	6687	188	6.42 (4.22-9.79)	<0.001	0.709
BMI	24-27.9	2840	188	4.22 (2.29-7.78)	<0.001	
BMI	>=28	636	54	16.24 (2.18-121.20)	0.007	
SBP	<140	8950	313	5.65 (3.90-8.18)	<0.001	0.976
SBP	>=140	1213	117	4.77 (2.13-10.68)	<0.001	

Note: HRs compare the high-persistent trajectory group with the low-ascending trajectory group within each subgroup. Subgroup results are exploratory because events were limited within some strata and no formal multiplicity adjustment was applied. Abbreviations: BMI, body mass index; CI, confidence interval; HR, hazard ratio; SBP, systolic blood pressure; TyG, triglyceride-glucose.

Table S7. Schoenfeld residual test for the proportional hazards assumption.

Term	Median P value	Range of P values
Global test	0.001	<0.000-0.002
TyG trajectory group	<0.001	<0.001-<0.001
Age	0.012	0.011-0.016
Sex	0.822	0.799-0.845
BMI	0.258	0.160-0.668
SBP	0.819	0.748-0.941
DBP	0.389	0.354-0.424

Term	Median P value	Range of P values
Uric acid	0.337	0.290-0.412

Note: Results summarize Schoenfeld residual tests across the five imputed datasets. Median and range of P values are reported. Abbreviations: BMI, body mass index; DBP, diastolic blood pressure; SBP, systolic blood pressure; TyG, triglyceride-glucose.

Table S8. Five-year restricted mean survival time analysis by TyG trajectory group.

TyG trajectory group	N	Events	5-year RMST (95% CI), years	Difference from G1 (95% CI), years
G1 low-ascending	4,272	50	4.98 (4.97 to 4.99)	Reference
G2 moderate-ascending	4,159	183	4.86 (4.83 to 4.88)	-0.12 (-0.15 to -0.09)
G3 high-persistent	1,732	197	4.56 (4.49 to 4.62)	-0.42 (-0.49 to -0.36)

Note: RMST was calculated from the post-landmark time origin. Differences are calculated relative to G1. Abbreviations: CI, confidence interval; RMST, restricted mean survival time; TyG, triglyceride-glucose.

Table S9. Formal comparison of time-dependent AUCs.

Time	Compared models	ΔAUC (95% CI)	P value
3 years	Model B - Model A	0.023 (0.012 to 0.034)	<0.001
3 years	Model C - Model A	0.039 (0.023 to 0.054)	<0.001
3 years	Model C - Model B	0.016 (0.006 to 0.027)	0.003
5 years	Model B - Model A	0.012 (0.002 to 0.023)	0.021
5 years	Model C - Model A	0.020 (0.007 to 0.032)	0.003
5 years	Model C - Model B	0.007 (-0.001 to 0.016)	0.093

Note: Model A was the base clinical model including baseline age, sex, BMI, SBP, DBP, and uric acid. Model B was Model A plus baseline TyG. Model C was Model A plus TyG trajectory group. ΔAUC indicates the difference in time-dependent AUC between models. Abbreviations: AUC, area under the curve; BMI, body mass index; CI, confidence interval; DBP, diastolic blood pressure; SBP, systolic blood pressure; TyG, triglyceride-glucose.

Table S10. Restricted cubic spline analysis by TyG trajectory group.

TyG trajectory group	P for overall association	P for nonlinearity
G1 low-ascending	0.047	0.376
G2 moderate-ascending	0.347	0.306
G3 high-persistent	0.369	0.345

Note: Restricted cubic spline analyses were performed within TyG trajectory groups. No clear evidence of nonlinearity was observed. Abbreviations: TyG, triglyceride-glucose.
